# A brief history of dopamine prediction errors

**DOI:** 10.3389/fncom.2026.1825622

**Published:** 2026-05-07

**Authors:** Biru B. Dudhabhate, Kauê M. Costa

**Affiliations:** Department of Psychology, University of Alabama at Birmingham, Birmingham, AL, United States

**Keywords:** associative learning, dopamine, learning theory, prediction error, reinforcement learning, reward, temporal difference learning, value

## Abstract

Dopamine signaling has become closely associated with reward prediction errors (RPEs)–the difference between expected and experienced value. Although not without controversy, the dopamine RPE hypothesis is one of the most influential ideas in neuroscience. This review briefly summarizes its origins, empirical foundations, and theoretical development. We begin with early psychological studies which demonstrated that prediction errors, broadly defined, are central drivers of learning. These experiments inspired mathematical models that formalized associative learning rules and informed the development of reinforcement learning algorithms for artificial learning, including the influential temporal difference learning (TDRL) framework, where learning is guided by prediction errors in value or reward. These theoretical proposals converged with neuroscience through the landmark discovery that midbrain dopamine neurons show activity patterns that are strikingly similar to the RPEs proposed in TDRL. The idea that this unique neuronal population, already implicated in several behavioral processes and brain disorders, could encode a computational variable central to reinforcement learning algorithms was a major conceptual shift, and provided a strong framework that allowed for rigorous hypothesis testing. Over the past three decades, increasingly sophisticated experiments have both replicated the core dopamine RPE finding across distinct experimental contexts and revealed important deviations from the canonical model predictions. These exceptions have sparked ongoing debate about how the hypothesis should be enhanced, revised, or replaced. The history of the dopamine RPE hypothesis is a quintessential example of how the integration of theory and experiments can drive progress in neuroscience and offers a template for theoretical–experimental synthesis.

## 1 Introduction

The stoic philosopher Epictetus pointed out that it is impossible for someone to learn what they already know (Epictetus, 2022). While this was meant to highlight the importance of intellectual humility, it captures the fundamental observation that learning requires the violation of preconceived expectations about the world. It is now well accepted that learning relies on the ability to perceive and properly reconcile the differences between prediction and experience, or “prediction errors”. While there is much controversy about how the brain computes prediction errors and what types of information are considered across different neural systems, there is general agreement that they define the updates in knowledge that drive learning.

Different types of prediction errors are implicated in learning about external associations (associative learning), understanding the effects of self-generated actions (reinforcement learning), shaping perception (predictive coding in sensory perception), and allowing for adaptive motor control (sensorimotor prediction errors) (Rescorla and Wagner, 1972; Sutton and Barto, 1998; Glimcher, 2011; Spratling, 2017; Popa and Ebner, 2019). This concept extends beyond the realm of neurobiology and behavioral sciences and is also central to modern computer science and artificial intelligence. These learning principles transcend the nature of the agent—whether it is biological or artificial—and can be generally applied to any system that acquires and organizes information to guide future decisions.

Many learning models, especially those applied to artificial intelligence, define learning as the update of a value function, or the estimation of all current and future rewards (desired outcomes) and punishments (undesired outcomes), discounted by factors like time delay. This type of learning depends on the difference between expected and experienced value, or reward prediction errors (RPEs). In the late 20th century, it was discovered that the activity of midbrain dopamine neurons very closely mimicked the dynamics of these RPEs (Schultz et al., 1997). This discovery revolutionized the field, changing the relationship between computational and experimental learning research (Schultz, 2007; Zhang et al., 2025).

Here, we briefly review the history of the dopamine RPE hypothesis, from the origins of the prediction error concept in early experimental psychology and learning theory, the theoretical development of learning mechanisms over the course of the 20th century, the experimental and computational advances that established this hypothesis, the impact it had on neuroscience and associated fields, and the current challenges that may overturn this paradigm. This review aims to be an accessible introduction to the history of this topic, and to invite readers to think about how the interaction between theory and experimentation shaped the current neuroscience research landscape.

## 2 The origins of prediction errors in learning theory

While there were important preceding and parallel studies (James, 1890; Twitmeyer, 1905), the rise of modern learning theory research is often dated to the late 19th and early 20th century, with, among other foundational work, Ivan Pavlov’s description of classical conditioning and Edward Thorndike’s law of effect. Pavlov demonstrated that a neutral stimulus (cue) paired with an unconditioned stimulus (outcome with motivational relevance, like food) can elicit a conditioned response (learned behavior related to the unconditioned stimulus) (Pavlov, 1927). This established the concept of conditioning, i.e., the idea that animals learn to respond to stimuli in anticipation of the outcomes that they predict, and that this occurs according to a set of rules. For example, presenting a conditioned stimulus in the absence of its unconditioned stimulus leads to extinction of the conditioned response, if two stimuli are presented simultaneously and followed by an unconditioned stimulus, the cue with the most salience becomes a larger driver of conditioned responses (overshadowing), and if another cue is paired with a previously conditioned cue it can also acquire the conditioned response of that cue (second-order conditioning). This framework set the stage for the systematic exploration of the rules that govern the learning of associations between cues and outcomes.

Thorndike’s Law of Effect, inspired by experiments with cats solving complex puzzles (i.e., operant learning), proposed that animals learn by trial and error, where actions leading to positive or pleasant outcomes (rewards) became more likely to be repeated, and actions leading to negative or unpleasant outcomes (punishments) become less likely to be repeated (Thorndike, 1927). This basic rule became the foundation of reinforcement learning, establishing the seed of the idea that animal behavior is driven towards maximizing reward and minimizing punishment (Skinner, 1938, 1963). For such learning, most of the information about the world could be condensed into the “pleasantness” or desirability of the learned outcome.

These foundational studies relied on the motivational relevance of unconditioned stimuli, i.e., rewards and punishments, to drive behaviors based on the nature of these outcomes, like salivation in anticipation of food, which allows for the direct quantification of learning in trial-based tasks, as pairing of a cue or action with one outcome changes responding in the immediately following trial. This led many to define learning as the progressive change in observable behavior (Skinner, 1963). However, challenging the idea that reinforcement was the only driver of learning, work by Tolman, Brogden, Lubow, and others showed that learning can occur in the absence of direct reinforcement (Brogden, 1939; Tolman, 1948; Lubow, 1973). This included the demonstration that presentation of neutral stimuli and associations incidentally learned in free exploration could guide future behavior. In other words, learning can be latent, or behaviorally silent, and animals can form associations between non-reinforcing stimuli. These critical discoveries demonstrated that learning is not fully dependent on the direct reinforcement value, a topic to which we will return to later in this review.

Another feature that was unclear from initial experiments was whether learning was only dependent on temporal contiguity and order, as was initially assumed. This would mean that associations would be formed between cues or actions and an unconditioned stimulus, so long as they were presented close together in time. However, the discovery of blocking ruled out this hypothesis. First demonstrated by Leon Kamin in rats, blocking is the phenomenon where, if an animal has already undergone asymptotic conditioning to a cue, then overlaying a new cue does not lead to new learning (Kamin, 1968, 1969). In effect, the previous learning to one cue “blocks” any additional conditioning to new cues. Later work further demonstrated that changing the properties of the unconditioned stimulus, like by increasing or decreasing the amount of the reinforcer, or changing its sensory properties, can unblock learning (Rescorla and Colwill, 1983; Holland, 1988; Holland and Kenmuir, 2005). Therefore, if all properties of an unconditioned stimulus are entirely predicted by a cue, then that unconditioned stimulus does not support additional learning to another cue, but if something changes about the outcome, then the ability to drive learning is restored. The discovery of blocking was an important mark in the development of learning theory, as it cemented the proposal that it is the predictive relationship between cue and outcome that defines learning, and that the violation of predictions, or prediction error, is the major driver of learning.

## 3 Formalization of prediction errors in learning

Prediction errors can be expressed mathematically in a relatively straightforward manner, as the simple difference between prediction and experience, which inspired the development of new theoretical models of learning. This was very important, and several models were proposed in the 1970s and 80s that incorporated prediction errors as drivers, or strong determinants of learning. By far the most influential of those was the Rescorla-Wagner model (Rescorla and Wagner, 1972; Siegel and Allan, 1996; Holland and Schiffino, 2016; Soto et al., 2023). This model described the influence of prediction errors on learning by asserting that they determine how much a reinforcer can drive learning—the more unexpected the outcome, the more it can drive changes in the strength of its association to predictive cues. This model was readily able to explain blocking, as it predicts that when one cue consistently predicts the outcome, it lowers the prediction error available for learning about other cues, which limits their associative gain. It also explained why more salient cues and outcomes led to higher associative strength by postulating separate salience terms that multiplied the update induced by the experience of a given prediction error, which could explain phenomena like overshadowing (Pavlov, 1927).

The Rescorla-Wagner model profoundly influenced the study of associative learning by providing a formal, mathematically grounded framework for understanding how prediction errors drive changes in associative strength (Miller et al., 1995; McNally and Yau, 2023; Soto et al., 2023). Its introduction instigated a massive response from the field and ushered in a new era in learning research (Siegel and Allan, 1996; Soto et al., 2023). That said, despite its ability to explain key features of learning, the Rescorla-Wagner model has significant limitations, which were apparent at the time of its proposal. These limitations included the fact that there is no clear definition of what “associative strength” means aside from the relationship between unconditioned and conditioned responses, that the model has no definition of time and therefore could only be applied to trial-based tasks, and that it fails to explain some basic learning phenomena, like latent inhibition (where the presentation of a cue with no reinforcement reduces subsequent learning) (Lubow, 1973). Alternative models, like the Bush and Mosteller (Bush and Mosteller, 1951), Mackintosh (Mackintosh, 1975) and Pearce-Hall (Pearce and Hall, 1980) models, provided key contributions and explained some experimental observations that the Rescorla-Wagner model could not, but were not as influential. Arguably, the success of the Rescorla-Wagner model was due to a combination of conceptual simplicity, historical timing, and the fact that it explained several prediction error-based learning phenomena in a manner that could be easily applied across tasks and species (Costa and Schoenbaum, 2021; Soto et al., 2023).

## 4 From animal learning to machine learning

The latter point of the previous section is one of the reasons why the Rescorla-Wagner became so influential outside the field of experimental psychology (Soto et al., 2023), including the (at the time) fledgling field of computer science and artificial intelligence. The discoveries and mathematical formalizations of learning theorists were the foundations for the development of artificial learning algorithms (Neftci and Averbeck, 2019). One of the most successful of these algorithms was temporal difference reinforcement learning (TDRL), introduced by Sutton and Barto in the 1980s (Sutton and Barto, 1981, 1998; Sutton, 1988). TDRL can be conceptualized as an extension of the Rescorla-Wagner learning rule to real-time events. Instead of relying on trial-based structures, the TDRL model can establish predictions for any number of continuous “states,” including those representing cues and outcomes. To do so, TDRL makes an important conceptual assumption—that learning involves the creation and update of a *value* function, or the representation of how good (or bad) any current or predicted state is. Essentially, if an agent is in a given state, the value of that state is defined by the reinforcing properties of any potential experience within that state, like a reward (or what would be an unconditioned stimulus in classical learning theory), in addition to the value of all subsequent states that can be predicted when in that state. Critically, this predicted value is discounted by how long the agent must wait for the outcome, with higher values for states that are closer in time. The value of a state is therefore defined as the discounted sum of all current and predicted rewards. This means that, in a task where every time point is set as a state, the TDRL model is able to make real-time predictions and updates to the agent’s value function. Moreover, the TDRL algorithm compares value predictions not only with the current state value, but also the value of all future predicted states—hence the term *temporal* difference. Prediction errors in this model are therefore *reward* prediction errors (RPEs), because they are explicitly about value, and also *temporal* prediction errors, because they are defined by the timing of predictions.

By moving away from the nebulous concept of associative strength, collapsing learning into a scalar variable, and using real-time rules, TDRL was able to explain several phenomena that were not within the purview of the Rescorla-Wagner model, like second-order conditioning and how animals could learn to time their conditioned responses to best match the delivery of the unconditioned stimulus (Sutton and Barto, 1998; Ludvig et al., 2012). Critically, this algorithm was highly successful in unsupervised machine learning applications that went way beyond the classical Pavlovian and operant tasks used to test learning in animals. It was used to train algorithms to play complex games like backgammon and chess at a master level (Tesauro, 1994; Baxter et al., 1998), and is a cornerstone of several modern machine learning and artificial intelligence algorithms (Mnih et al., 2015; Silver et al., 2018; Alkam et al., 2025). TDRL is therefore a key example of how learning algorithms inspired by psychological principles can be applied to self-learning in artificial systems across multiple tasks.

## 5 The dopamine revolution: neurobiological evidence for reward prediction errors

In the mid-1990s, the prediction error-based learning rules that were first derived from experimental psychology experiments, formalized by learning theorists, and expanded and refined by computer scientists, would return to biology and establish itself in neuroscience. In a landmark paper, Schultz, Dayan, and Montague (Schultz et al., 1997) provided compelling evidence that a large fraction of midbrain dopamine neurons in behaving macaques had activity patterns that very closely mimicked the RPEs proposed in TDRL models of learning. Briefly, most recorded primate dopamine neurons (55 to 80% in those experiments) increased their firing whenever an unexpected reward or reward-predicting cue was presented, decreased their firing when an expected reward was omitted, and did not change their firing whenever a fully expected reward was presented, almost exactly as expected from a TDRL RPE representation. The hypothesis put forth was that the brain executes a form of TDRL computation to drive adaptive learning, and that dopamine was a key RPE reinforcement signal. This bold proposal was built on more than a decade of careful recording experiments (Schultz, 1986; Schultz et al., 1993; Mirenowicz and Schultz, 1994, 1996) and parallel theoretical developments (Montague et al., 1996). Moreover, the sheer impact of how well the activity of the neurons appeared to match with the proposed theory was remarkable, and still impresses newcomers to the field. Very few neural correlates have such a striking “first glance” face validity as the dopamine RPE, and it’s effect is rivalled perhaps only by the observation of hippocampal place cells (O’Keefe and Dostrovsky, 1971).

The dopamine RPE hypothesis sparked a major conceptual revolution in the field. Part of what made it so impactful was that the encoding of RPEs by dopamine neurons made sense from a mechanistic point of view. Dopamine, a catecholamine neuromodulator, was already implicated at the time in synaptic plasticity, reward and learning (Robbins and Everitt, 1992; Costa and Schoenbaum, 2022). Dysfunctions in dopamine signaling had also already been extensively demonstrated in diseases characterized by pathological learning, like drug addiction (Koob, 1992; Nestler, 1992) and schizophrenia (Carlsson, 1988). Indeed, dopamine dysfunction is observed in almost all neurological and psychiatric disorders (Costa and Schoenbaum, 2022), and the idea that there could be a computational principle that unified at least some component of these different diseases within a common theoretical framework was very attractive.

Dopamine RPEs quickly became a centerpiece of computational psychiatry, the field by which mental health diseases are explored as disorders of particular computational processes. For example, dopamine’s role in addiction could be explained as unbounded RPEs (Redish, 2004; Lehmann et al., 2025), and it’s role in schizophrenia as maladaptive prediction errors that create spurious associations (Millard et al., 2022). Of course, there is extensive debate about whether any of these propositions are true, but the key point is that these propositions can only exist, and potentially be falsified, in a world where there is a clear computational hypothesis for the behavioral role of dopamine.

The main advantage of having a strong formal hypothesis is that experimenters can design experiments targeted towards falsifying specific predictions from that proposal, and test whether the assumptions hold across experimental contexts (Costa and Schoenbaum, 2021). In the years after the seminal dopamine RPE study, the finding that most dopamine neurons, and dopamine release in certain target regions, convey an RPE-like signal was indeed replicated across different tasks (Tobler et al., 2003; Bayer and Glimcher, 2005), species (Cohen et al., 2012; Hart et al., 2014; Kishida et al., 2016), and recording methods of dopamine activity (Pessiglione et al., 2006; Day et al., 2007; Patriarchi et al., 2018), with critical exceptions that we will address in the next section.

The development of genetic tools and optogenetic manipulation also allowed for the causal test of this hypothesis. In an “instant classic” study, Steinberg and colleagues demonstrated that optogenetic stimulation of dopamine neurons in rats was sufficient to unblock learning (Steinberg et al., 2013). This unblocking effect was a striking finding that experimentally confirmed a key prediction of the proposal that prediction errors drive learning, coming full circle to the classical Kamin blocking experiments discussed earlier in this review. Similarly, inhibition of dopamine neurons was shown to mimic negative prediction errors (Chang et al., 2016, 2018), and these tools also provided insight into the cell type-specific circuitry that shapes the computation of RPE-like signals (Cohen et al., 2012; Eshel et al., 2015, 2016).

## 6 Beyond the classical RPE: recent developments, challenges, and new directions

The dopamine RPE hypothesis is so strong, and its effects are so widespread that it is nearly impossible to work in the field without addressing it in one way or another, and the finding that dopamine signals can be very similar to RPEs is likely one of the most replicated findings in all of neuroscience. However, there are also several findings about dopamine function that cannot be fully explained by the standard TDRL framework (Costa and Schoenbaum, 2025; Zhang et al., 2025).

First among those is the finding that dopamine neurons are not all the same. Over the course of the first two decades of this century, it was progressively demonstrated that dopamine neurons that project to different regions have different genetic profiles and activity patterns, including many that do not look like RPEs (Roeper, 2013; Lammel et al., 2014; Menegas et al., 2017; Farassat et al., 2019; Poulin et al., 2020; Azcorra et al., 2023; Knowlton and Canavier, 2023). This diversity was already foreshadowed in the first proposal of the TDRL hypothesis as, again, only a fraction of dopamine neurons showed RPE-like activity patterns (Schultz et al., 1997). Perhaps this should not have been much of a surprise, as dopamine is a neurotransmitter like any other and it is natural that it should have distinct functions in distinct systems. Such a simplistic heuristic would be unthinkable for most other neurotransmitters, and perhaps only became popular because of the influence of the RPE hypothesis. With the research of the last two decades, it seems now that the dopamine pathway where RPE-like patterns are most evident is the mesolimbic pathway, or dopamine neurons in the ventral tegmental area that project to regions of the ventral striatum, especially the nucleus accumbens (Hart et al., 2014; Menegas et al., 2017; Mohebi et al., 2024; Costa et al., 2025a).

But beyond pathway-specific differences, even in the same type of dopamine neurons that seem to show classical RPEs, there are still discrepancies from the classical TDRL model that were revealed by the progressive testing of the RPE hypothesis in different tasks. Indeed, if one records dopamine signals in tasks that can be solved by a simple TDRL model, then the signals mostly conform to a simple TDRL model. It is only when animals engage in behaviors that go beyond the purview of TDRL that the limits of the model for explaining dopamine function become clear. This is because the initial version of the TDRL model has many assumptions which limit its capacity to explain complex behaviors, like that learning involves the update of a single value function, and that updates to this function only occur through directly experienced value changes (Schultz et al., 1997; Sutton and Barto, 1998; Costa and Schoenbaum, 2025). Conditions that cannot be explained within these boundaries require at least extensions and modifications to the classical TDRL framework.

For example, it was shown in 2013 that dopamine release in the nucleus accumbens ramps up as animals approach a fully predicted reward, which should not happen according to classical TDRL RPE definitions (Howe et al., 2013). This sparked heated debate, which still continues, about what exactly these ramps mean, as these persistent responses seemed to better reflect value or motivation rather than RPEs (Hamid et al., 2015; Mohebi et al., 2019; Guru et al., 2020; Kim et al., 2020; Costa et al., 2025b; Floeder et al., 2025; Priestley and Akam, 2026). However, from a theoretical viewpoint, a TDRL rule can explain these ramps by simply assuming a convex transformation of proximity to the goal—a straightforward tweak to the classical model (Gershman, 2014). This is one example of how the basic TDRL model can be modified or updated to reconcile with experimental findings, without changing the core rules of the TDRL RPE.

Other observations that cannot be explained by classical TDRL RPEs include the discovery that dopamine neurons can respond to prediction errors in reward identity between rewards of similar magnitude (Lak et al., 2014; Takahashi et al., 2017; Stalnaker et al., 2019), that dopamine signaling determines learning about reward features (Chang et al., 2017; Keiflin et al., 2019), that different dopamine neurons seem to track changes in different reward-related variables, like movement kinematics and reinforcement history, during more complex reward-based tasks (Engelhard et al., 2019), and that the devaluation of a specific reinforcer immediately reduces dopamine RPE responses to that stimulus (Papageorgiou et al., 2016). In recent developments, several groups have reconciled these observations by proposing the existence of several parallel value functions and state representations encoded by different dopamine neurons (Millidge et al., 2023; Takahashi et al., 2023; Lee et al., 2024). Therefore, the TDRL RPE hypothesis of dopamine signaling is not static, and this core proposal can, and has been, updated to account for new experimental observations (Costa and Schoenbaum, 2025; Zhang et al., 2025).

That said, there are cases where reconciliation seems impossible. These findings require fundamentally different computational frameworks. The strongest challenges to the dopamine RPE hypothesis comes from studies that explicitly tested the fundamental assumptions of TDRL in relation to value. These include discoveries that dopamine mediates learning in the absence of any reinforcement (Sharpe et al., 2017; Kutlu et al., 2022; Costa et al., 2025a) and that it can control the associability of stimuli (Morrens et al., 2020; Kutlu et al., 2021, 2022). These findings, among many others not listed here, fundamentally challenge the idea that dopamine signals only a value-based RPE. Alternative proposals therefore must go beyond the confines of TDRL. Current examples include proposals that dopamine encodes salience (Matsumoto and Hikosaka, 2009; Kutlu et al., 2021), causal inference (Jeong et al., 2022), or prediction errors about features beyond value (Takahashi et al., 2017; Gardner et al., 2018; Langdon et al., 2018; Corlett et al., 2022; Costa et al., 2025a).

The latter proposal harks back to the work by Tolman and others on learning that is independent of direct reinforcement (Tolman, 1948), and also builds on developments in computational models where learning involves not just creating a value function, but building a model of the transition probabilities between observable and latent states, also known as “model-based learning” (Langdon et al., 2018; Akam and Walton, 2021; Costa and Schoenbaum, 2025; Costa et al., 2025a). In this framework, dopamine would still signal prediction errors, but instead of just value, these prediction errors would incorporate information that define the states that comprise the animal’s internal model. This is interesting because it retains the idea of dopamine signaling prediction errors, but errors that are richer than previously thought, and inspires a search for what types of information may be used to compute these errors.

## 7 Conclusion

Prediction errors as a concept proved to be widely applicable to psychology, machine learning and neuroscience, where the idea that a specific type of prediction error, a TDRL RPE, is instantiated by dopamine signaling has inspired decades of discovery. The field is currently ablaze with ardent debate on the topic, and we are likely in the transition period to a new paradigm in which the TDRL framework will be significantly improved or abandoned altogether. A detailed discussion of the evidence in favor of or against any of the updates or the alternative proposals to TDRL is beyond the scope of this review, but what we want to impress on the reader is the importance of the dopamine RPE hypothesis as the benchmark and inspiration for these recent developments. Even if ultimately abandoned, the historical importance of the dopamine RPE hypothesis is unquestionable. It is the quintessential example of successful long-term synergy between theoretical and experimental neuroscience, and is an example to be followed by other neuroscience fields where strong normative hypotheses are still lacking.
